# Hemorrhagic stroke in children caused by *Bothrops marajoensis* envenoming: a case report

**DOI:** 10.1186/s40409-015-0052-5

**Published:** 2015-12-14

**Authors:** Pedro Pereira de Oliveira Pardal, Augusto Cezar Jennings da Silva Pinheiro, Cristiane Tarcis Cunha Silva, Paulo Roberto Silva Garcez Santos, Maria Apolônia da Costa Gadelha

**Affiliations:** University Hospital João de Barros Barreto, Federal University of Pará (UFPA), Rua dos Mundurucus 4487, Guamá, 66073.000, Belém, PA Brasil; School of Medicine, Federal University of Pará (UFPA), Belém, PA Brazil

**Keywords:** Hemorrhagic stroke, Sequel, *Bothrops marajoensis*, Marajó Island

## Abstract

According to the World Health Organization, snakebites are considered neglected diseases. *Bothrops*, the genus most frequently implicated in envenomations in Brazil, includes the species *B. marajoensis* Hoge, 1966, part of the complex *B. atrox*, which is found in the savannas of Marajó Island, Pará state, Brazil, a region that presents scarce epidemiological data. This work reports the first case of hemorrhagic stroke in a child, attributed to delayed medical care after snakebite envenoming by *Bothrops marajoensis* in Anajás city, Marajó Island, Pará, Brazil, which led to permanent hemiplegia as a sequela.

## Background

Snakebites were included by the World Health Organization on the list of neglected diseases [1, 2]. An incidence of 5 million envenomings annually worldwide is estimated, with the highest incidence in developing countries with agricultural activities [3]. In Latin America there are approximately 129,084 cases annually, with the vast majority being caused by species of the family Viperidae [4]. In Brazil, from 2011 to 2014 there were 112,249 accidents, 72 % caused by *Bothrops* [5].

The genus *Bothrops* has neotropical distribution, with the species *B. marajoensis* Hoge, 1966 [6] being found in the savannas of Marajó Island, Pará state, Brazil [7]. This species is part of the complex *B. atrox*, popularly known as “jararaca”, “surucucurana”, “combóia” and even by “surucucu” [8]. The *Bothrops* are implicated in a large number of envenomings in the Brazilian Amazon, where they affect mainly rural areas and are considered a public health problem. The epidemiological data on this species are scarce and underestimated in this region [9, 10].

Snake venom is a complex mixture containing biologically active peptides and proteins that can cause local inflammatory responses and changes of blood coagulation due to defibrination, disseminated intravascular coagulation and thrombocytopenia resulting in partial or complete blood incoagulability, leading to systemic bleeding that may provoke a hemorrhagic stroke [11–13]. But in children this picture is poorly described, which encouraged us to report the first case associated with snakebite envenoming by *B. marajoensis* [14, 15].

## Case presentation

**Fig. 1:**
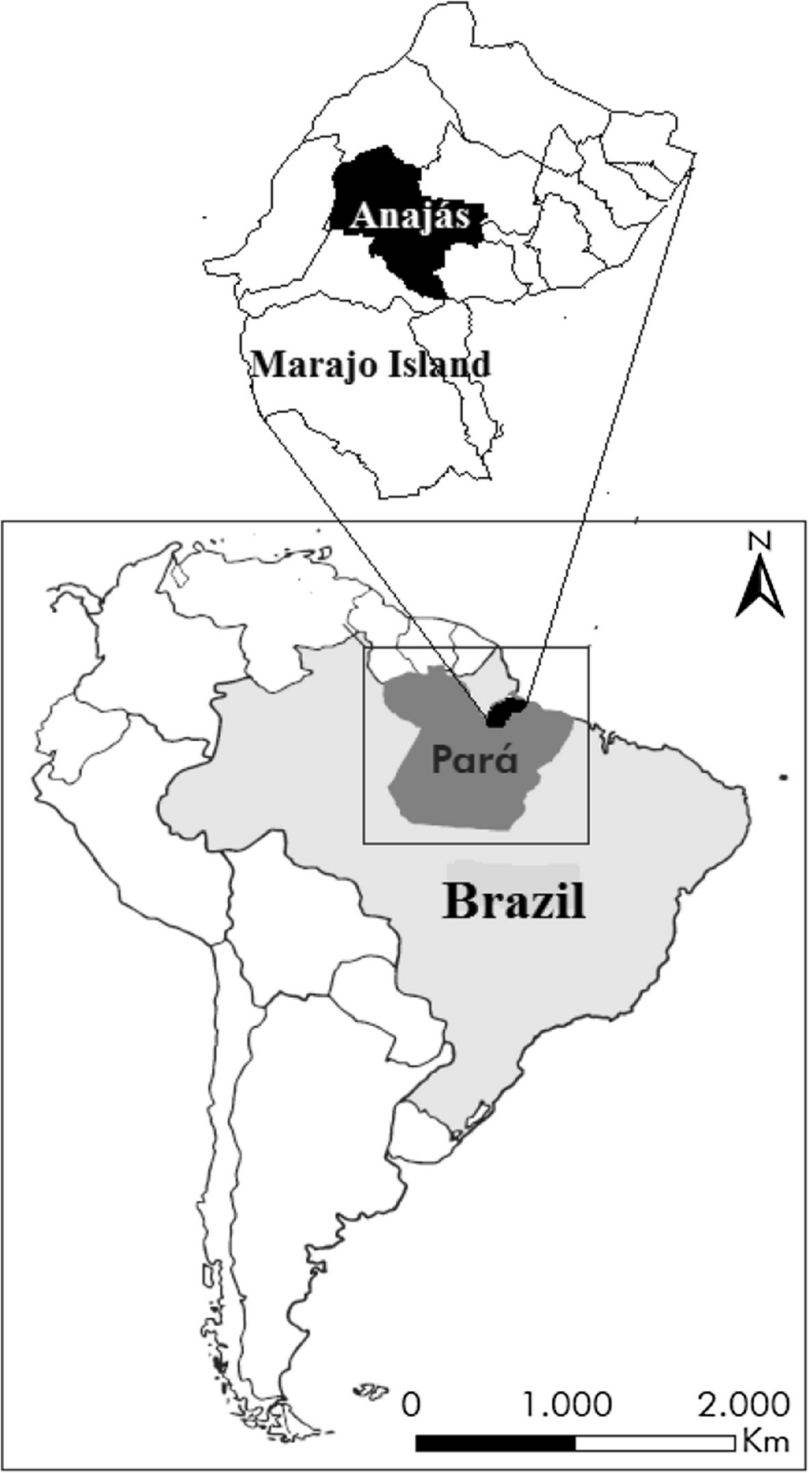
Map of Marajó Island in the state of Pará, highlighting the city of Anajás, the envenomation site

**Fig. 2:**
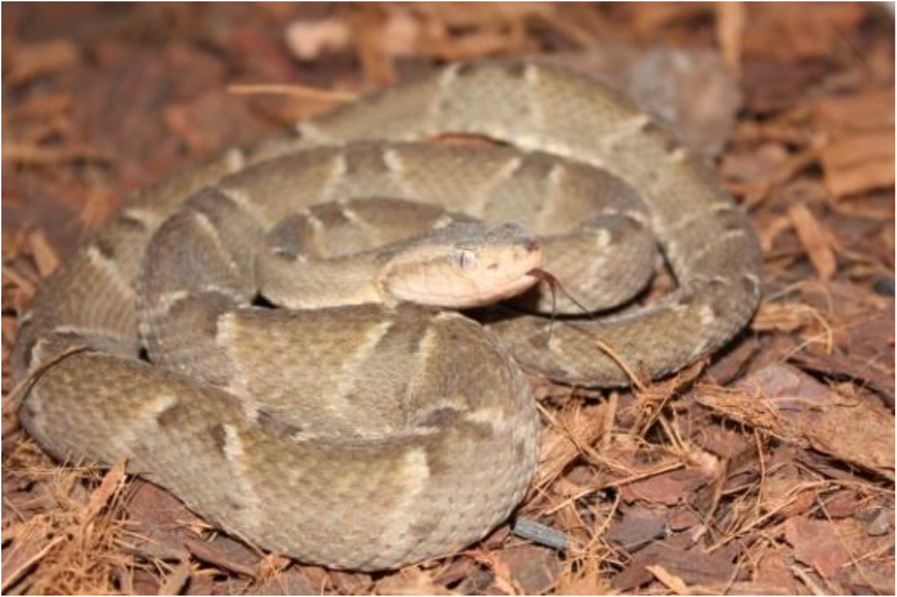
*Bothrops marajoensis* of the Marajó archipelago. Photo by Leandro Oliveira

A male child younger than 10 years old, from Anajás city, Marajó Island, Pará (latitude 00°59'14" and longitude 49°56'25") (Fig. 1), was bitten in an attack by a snake identified as *B. marajoensis* Hoge, 1966 [6] (Fig. 2), in the plantar region of the right foot, on July 14, 2013, while harvesting açaí.

After the bite, the patient reported local pain then local edema. His parent took him by motorized boat for medical assistance in Anajás. During the trip the boat malfunctioned, which delayed medical care for 25 h. During transport, the child became somnolent and stopped communicating. Upon admission at the hospital, he presented with edema on the entire right leg, ecchymosis, blisters, hematuria and a comatose state, and was classified as severely poisoned.

The child received all four vials of antibothropic antivenom available in the service unit. A transfer to Belém city, the capital of Pará state, was requested, and effected 2 days after to the Emergency Department Mario Pinotti by helicopter. He was admitted with the aforementioned clinical manifestations and the same severity, with eight antivenom vialshaving been infused to complete the number of ampoules required due to the gravity of the case.

**Fig. 3:**
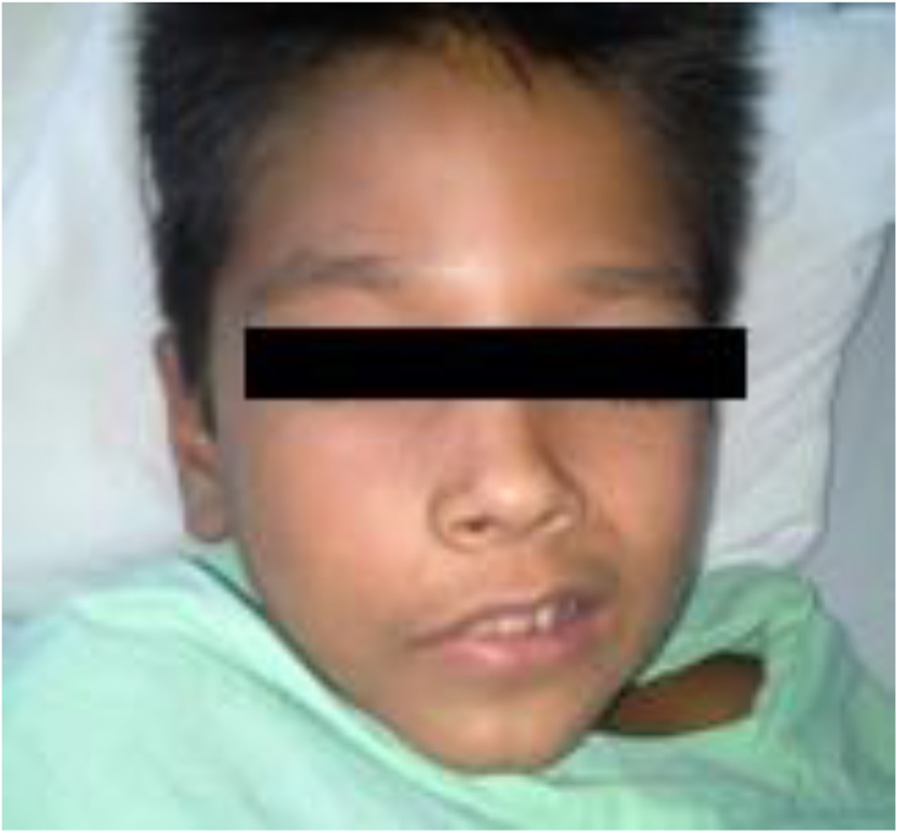
Labial commissure deviation to the left side

He was transferred on 19 July to the University Hospital João de Barros Barreto in Belém, where he was admitted comatose, with right hemiplegia, labial commissure deviation to the left side (Fig. 3), heart and lung auscultation without abnormalities, swelling from the foot to the knee, blisters, normal urinary color and volume and signs of infection at the bite site.

**Fig. 4:**
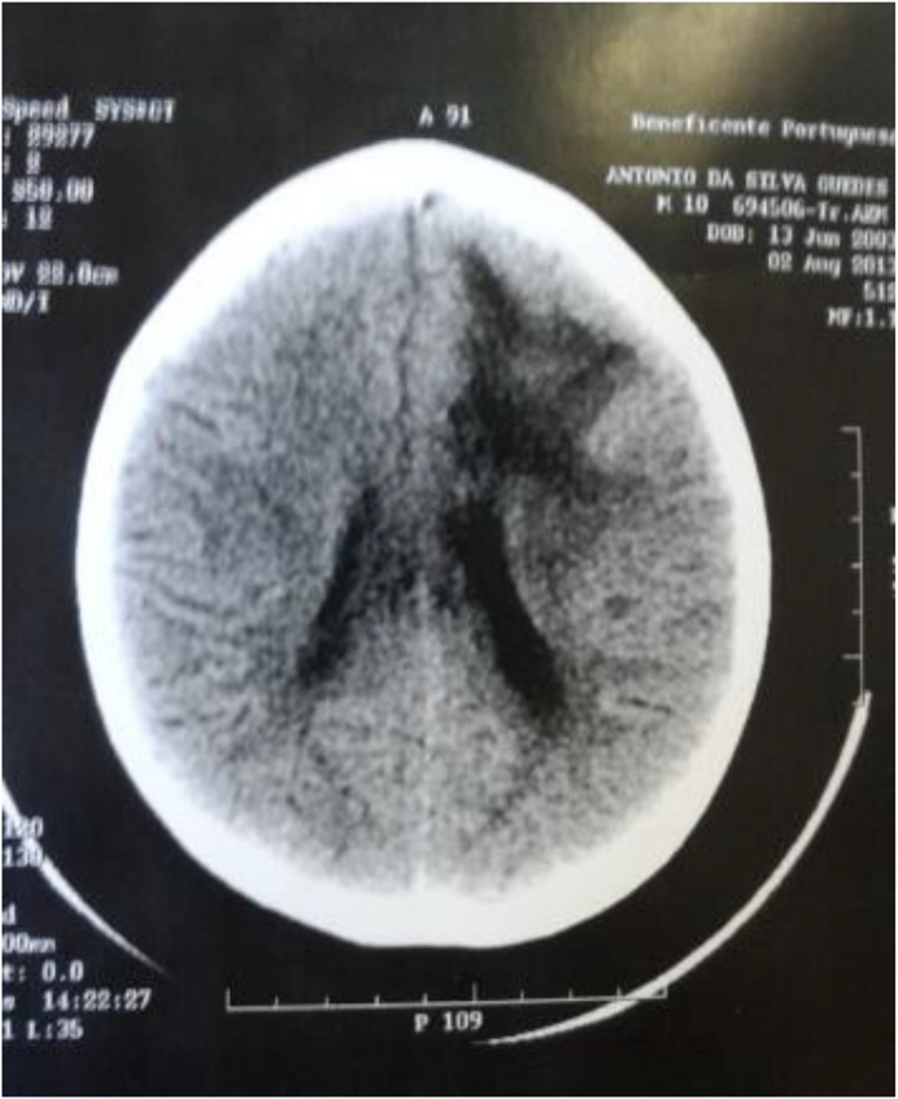
Cranial computed tomography showing intracerebral hemorrhage in the right frontal area

He was evaluated by a neurologist who requested laboratory tests (Table 1) and acomputed tomography of the brain that showed hemorrhagic lesions (Fig. 4). Conservative therapeutics and antibiotic therapy were applied. During the 19 days of hospitalization, the patient received medical and physiotherapist follow-up, receiving medical discharge with sequelae including right flaccid hemiplegia.

**Table 1:** Laboratory tests conducted at the Hospital João de Barros Barreto on July 19, 2013

Exams	Results	Reference values
Hemogram	3.61 millions/mm^3^	4.4 to 5.9 millions/mm^3^
Hemoglobin	10.3 g/dL	13.8 to 18.0 g/dL
Hematocrit	31.1 %	40 to 52 %
Leukogram	6.54/mm^3^	5,000 to 10,000/mm^3^
Segmented	90 %	40 to 75 %
Platelet count	121,000/mm^3^	130,000 to 400,000/mm^3^
APTT^a^	20.3 s	25 to 34 s
Urea	27 mg/dL	10 to 40 mg/dL
Creatinine	0.5 mg/dL	0.4 to 1.2 mg/dL
Creatine kinase (CK)	915 lU/mL	fem <145 ǀ male < 171

^a^Activated partial thromboplastin time

## Discussion

A case of hemorrhagic stroke in a child associated with envenoming by *B. marajoensis* in Anajás city, Marajó Island, is herein reported. This type of accident in the pediatric age group is rarely described in the literature, but is more frequently reported in adults. Mosquera, Idrovo and Tafur [16] reported an incidence of 2.6 % of strokes in *Bothrops* victims in Ecuador, where as Thomas et al. [17] found 2.3 % in Martinique. Santos-Soares et al. [18] and Machado et al. [19] also reported cases of stroke following snakebites in Brazil.

*Bothrops*is the main genus involved in snakebites in Brazil [20]. In the Amazon, ophidic accidents are attributed mostly to *B. atrox* species complex, represented in the archipelago of Marajó by *B. marajoensis* [21, 22]. In all 144 cities of Pará state in the 2012–2014 period, there were 14,842 snakebite accidents in total; of these, 86.84 % by *Bothrops*. Two thousand two hundred and seventy-one (2271, i.e., 15.3 %) of these envenomings occurred in the 16 cities of Marajó Island [5].

The venom of snakes of the genus *Bothrops* are complex mixtures of proteins and peptides that present various toxic activities leading to inflammatory, coagulant and hemorrhagic effects [12, 23]. Symptoms of envenoming are determined by the type of venom, the agent, the severity of the accident and by the conditions inherent to the victim, which may include local manifestations (pain, swelling and redness) and systemic manifestations (bleeding). Complications of envenoming with hemorrhagic stroke are more evident in patients with preexisting risk factors such as cardiovascular disease, particularly systemic hypertension, which was not detected in this case [19, 24].

The accident occurred in the country side, when the child was descending an açaí palm tree (*Euterpe oleracea*), which bears a berry much appreciated by people and is a very popular food supplement. Snakebite is a common and frequently devastating occupational and environmental disease, especially in rural areas of tropical developing countries [25].

The accident site is about 7 hours away from medical care. Using a motorboat, however, the patient was only able to reach the hospital of the municipal headquarters 25 h after the event, due to engine failure on the boat that was transporting him. This is a common situation in the region, as the medical care is distant from where accidents normally occur. The precarious means of transport greatly aggravates the clinical condition of the victims. It is recommended by the Brazilian Ministry of Health that the initial care of the patient bitten by venomous animals should be obtained as soon as possible, with the number of vials based on the severity of the accident [20]. In this case, the initial treatment should consisted of 12 ampoules of antivenom. In Brazil, the antivenom used is liquid and requires electricity for its conservation, which delays treatment, since accidents occur in rural areas [20]; hence, if we had lyophilized serum in these regions, we believe that similar cases would not occur.

Hemorrhagic stroke is characterized by bleeding into brain tissue, the cerebellum, or brain stem, usually by rupture of a small vessel, causing the overflowed blood under pressure in the brain tissue to lead to clinical manifestations that depend on the location and the specific extent of bleeding [26]. The signs and symptoms observed in the present case included an acute onset without history of trauma and progressed to drowsiness and coma. In the United States, half of pediatric strokes are caused by bleeding in association with several other causes, but without references to envenoming by snakes [27]. In Brazil, Bucaretchi et al. [28] did not find any cases of stroke in 322 children envenomed by snakes. In our case, the clinical examination revealed right hemiplegia and deviation of the labial commissure to the left, while the cranial computed tomography scan showed bleeding lesion in the right frontal area, which characterized an acute neurological presentation of hemorrhagic stroke [16].

Intracerebral hemorrhage after snakebite is related to the occurrence of severe coagulopathy, which is not shown in this case due to the fact that the tests had been administered belatedly [29]. Pardal et al. [9] showed that *B. atrox* was responsible for 13.3 % of blood incoagulability while Ribeiro et al. [30] reported four cases of intracerebral hemorrhage caused by *Bothrops* that presented coagulopathy. These brain bleeding disorders have been linked with incoagulability and low platelet count associated with brain capillary endothelial damage caused by hemorrhages and possibly by other toxins found in the snake venom [12, 31]. Other laboratory exams such as blood count showed anemia, while total creatine phosphokinase appeared five times higher than the reference value, which is related to the inflammatory process at the bite site and brain injury in this case. This enzyme is mainly used to assess changes in the heart muscle, skeletal muscle and brain [31].

## Conclusion

This is the first case report of hemorrhagic stroke in a child attributed to delayed medical care after snakebite envenoming by *Bothrops marajoensis* on the island of Marajó, which led to permanent hemiplegia as a sequela.

## Consent

Written informed consent was obtained from the legal guardian of the patient for publication of this case report and accompanying images.

## Ethics committee approval

This manuscript was approved by the Research Ethics Committee of the University Hospital João de Barros Barreto, document number 41157015.3.0000.0017.

## References

[1] World Health Organization. In: Neglected tropical diseases: Snakebite. http://www.who.int/neglected_diseases/integrated_media_snakebite/en/. Accessed 15 Apr 2015.

[2] Bochner R (2013). The international view of envenoming in Brazil: myths and realities. J Venom Anim Toxins incl Trop Dis.

[3] Chippaux JP (1998). Snake-bites: appraisal of the global situation. Bull World Health Organ.

[4] Gutiérrez JM (2014). Current challenges for confronting the public health problem of snakebite envenoming in Central America. J Venom Anim Toxins incl Trop Dis..

[5] Secretaria de Vigilância em Saúde. Sistema de Informação de Agravos de notificação (SINAN). Brasília. Ministério da Saúde do Brasil. 2015. http://dtr2004.saude.gov.br/sinanweb/tabnet/dh?sinannet/animaisp/bases/animaisbrnet.def

[6] Hoge, AR. "Preliminary account on Neotropical Crotalinae (Serpentes: Viperidae)." Mem. Inst. Butantan (1966)32:109–184

[7] Hoge AR, Romano AS (1973). Sinopse das serpentes peçonhentas do Brasil. Serpentes, Elapidae e Viperidae. Mem Inst Butantan.

[8] Wüster W, Golay P, Warrell DA (1998). Synopis of recent developments in venomous snake systematics. Toxicon.

[9] Pardal PP, Souza SM, Monteiro MR, Fan HW, Cardoso JL, França FO (2004). Clinical trial of two antivenoms for the treatment of *Bothrops* and *Lachesis* bites in the north eastern Amazon region of Brazil. Trans R Soc Trop Med Hyg.

[10] Lima ACSF, Campos CEC, Ribeiro JR (2009). Perfil epidemiológico de acidentes ofídicos do Estado do Amapá. Rev Soc Bras Med Trop.

[11] Gutiérrez JM, Meier J, White J (1995). Clinical toxicology of snakebite in Central America. Handbook of clinical toxicology of animal venoms and poisons.

[12] Gutiérrez JM (2002). Comprendiendo los venenos de serpientes: 50 años de investigaciones en América Latina. Rev Biol Trop.

[13] Shivanthan MC, Yudhishdran J, Navinan R, Rajapakse S (2014). Hump-nosed viper bite: an important but under-recognized cause of systemic envenoming. J Venom Anim Toxins incl Trop Dis..

[14] Bashir R, Jinkins J (1985). Cerebral infarction in a young female following snake bite. Stroke.

[15] Vale TC, Leite AF, Hora PR, Coury MI, Silva RC, Teixeira AL (2013). Bilateral posterior circulation stroke secondary to a crotalid envenomation: case report. Rev Soc Bras Med Trop.

[16] Mosquera A, Idrovo LA, Tafur A, Del Brutto OH (2003). Stroke following *Bothrops* spp. snakebite. Neurology.

[17] Thomas L, Chausson N, Uzan J, Kaidomar S, Vignes R, Plumelle Y (2006). Thrombotic stroke following snake bites by the “Fer-de-Lance” *Bothrops lanceolatus* in Martinique despite antivenom treatment: A report of three recent cases. Toxicon.

[18] Santos-Soares PC, Bacellar A, Povoas HP, Brito AF, Santana DLP (2007). Stroke and snakebite: case report. Arq Neuro-Psiquiatr.

[19] Machado AS, Barbosa FB, Mello GS, Pardal PPO (2010). Acidente vascular cerebral hemorrágico associado à acidente ofídico por serpente do gênero *Bothrops*: relato de caso. Rev Soc Bras Med Trop.

[20] Fundação Nacional de Saúde (2001). Manual de diagnóstico e tratamento de acidentes por animais peçonhentos.

[21] Amaral A (1924). On the biological differentiation of the neotropical species of snakes, *Bothrops atrox* (Linné 1758), *B. jararaca* (Wied 1924), and *B. jararacussu* (Lacerda 1884). Am J Trop Med Hyg.

[22] Antunes JF. Diversidade filogenética, distribuição geográfica e prioridades de conservação em jararacas sul-americanas (serpentes: Viperidae: *Bothrops* e *Bothrocophias*). Dissertação de Mestrado. Universidade de Brasília, 2012. http://repositorio.unb.br/bitstream/10482/11108/1/2012_JessicaFenkerAntunes.pdf

[23] Markland FS (1998). Snake venoms and the hemostatic system. Toxicon.

[24] Gutiérrez JM, Rucavado A, Escalante T, Lomonte B, Ângulo Y, Fox JW (2010). Tissue pathology induced by snake venoms: How to understand a complex pattern of alterations from a systems biology perspective?. Toxicon.

[25] Warrell DA (2010). Snake bite. Lancet.

[26] Zivin JA, Goldaman A (2005). Doença vascular cerebral hemorrágica. Tratado de Medicina Interna.

[27] Lynch JK, Hirtz DG, De Veber G, Nelson KB (2002). Report of the National Institute of Neurological Disorders and Stroke workshop on perinatal and childhood stroke. Pediatrics.

[28] Bucaretchi F, Herrera SR, Hyslop S, Baracat EC, Vieira RJ (2001). Snakebites by *Bothrops* spp in children in Campinas, São Paulo, Brazil. Rev Inst Med Trop São Paulo.

[29] Sprivulis P, Jelinek GA (1995). Fatal intracranial haematomas in two patients with brown snake envenomation. Med J Aust.

[30] Ribeiro LA, Albuquerque MJ, Pires de Campos VAF, Katz G, Takaoka NY, Lebrão ML (1998). Óbitos por serpentes peçonhentas no Estado de São Paulo: avaliação de 43 casos, 1988–93. Rev Ass Med Bras.

[31] Cantarow A, Trumper M (1975). Clinical Biochemistry.

